# Criteria for Occupational Health Prevention for Solar UVR Exposed Outdoor Workers-Prevalence, Affected Parties, and Occupational Disease

**DOI:** 10.3389/fpubh.2021.772290

**Published:** 2022-01-26

**Authors:** Marc Wittlich

**Affiliations:** Department Accident Prevention: Digitalisation-Technologies, Institute for Occupational Safety and Health of the German Social Accident Insurance, Sankt Augustin, Germany

**Keywords:** UV radiation, occupational health prevention, occupational safety and health, UV personal dosimetry, occupational disease, skin cancer

## Abstract

Non-melanoma skin cancer (NMSC) is the most common cancer in western countries. Legislative bodies and stakeholders like WHO and EU strongly promote protection against solar UVR, especially in workers. Occupational health prevention must be introduced as a strong instrument in workers protection also with regard to occupational disease issues. To date, criteria for both occupational health prevention and occupational disease are missing and the identification of risk groups has no metric basis. Here I report a criteria analysis based on the largest comprehensive data set of occupational ultraviolet radiation exposure of outdoor workers. With detailed research on occupation-specific dosimetric measurements of 45.000 measurement days in 176 occupations and sub-occupations, it is possible to map criteria for occupational health prevention specifically and to identify affected occupations. The number of employees affected can be elucidated worldwide. For the first time, a direct link to retrospective occupational disease criteria could be established. Of the 176 occupations and sub-occupations selected for this work, 153 (=87%) exceed the criterion for occupational health prevention and thus need special attention. This includes all occupations with annual exposures of more than 150 SED. Employment figures for the EU and the world yield the total number of affected workers to be 36.1 million and more than 500 million, respectively. These new criteria for occupational health prevention are valid and in good agreement with international research on limit values by WHO and ICNIRP. If applied correctly and consistently, these criteria can prevent occupational disease. It will be possible to identify occupations and sub-occupations that have an urgent need for prevention to avoid chronic skin damage leading to cancer. This research serves as a basis for policy making and clinical risk identification, as well as for daily practice of occupational physicians and employers responsible for risk assesment.

## Introduction

Non-melanoma skin cancer (NMSC) is the most common cancer in western countries. NMSC includes squamous cell carcinomas (SCC), including actinic keratoses (AK) and basal cell carcinomas (BCC). For the same exposure situation, the extent to which the population is affected depends, in part, on the distribution of skin types according to the Fitzpatrick scale (1), which describes and classifies the tolerance of the skin to solar ultraviolet radiation (UVR). In addition to the benefits for dermatology, the classification according to the Fitzpatrick scale has been used directly in prevention, for example in workers protection evaluation criteria (2, 3).

The WHO attaches great importance to NMSC by UVR. It has been reported that 65–90% of all skin cancers are attributable to solar UVR exposure (4, 5), even by collaboration centers (CC) of the WHO like the Cancer Council Australia (www.cancer.org.au). NMSC occurs frequently, but death is unlikely. Nevertheless, it has a considerable impact on the quality of life. Incidence rates continue to rise worldwide for both SCC and BCC (6, 7). This is also evidenced by the DALYs (Disability adjusted life years) (8), especially with regard to NMSC. From 2000 to 2019, it almost doubled from 0.032 to 0.06% (of total DALYs). It can be assumed that the DALYs for NMSC are underestimated, as reporting by both those affected and authorities is weak.

The particular importance of the issue has clearly increased, especially at the European level. The Beating Cancer (BECA) Committee of Members of the European Parliament (MEPs) set up by the European Parliament is dealing with the content of Europe's Beating Cancer Plan (9). It was stipulated that the incidence of cancer should be reduced by 30%. The BECA committee has also determined that occupational skin cancer is the priority target of the activities.

The prevention of work-related health hazards and the preservation of employability are of great importance and a task for the society as a whole. Occupational health prevention (OHP) is an essential part of occupational health and safety measures. The aim of OHP is the prevention and early detection of work-related diseases. It is also intended to contribute to maintaining employability and the further development of occupational health and safety. In Germany, OHP is based on the Occupational Health and Safety Act (10) and the Ordinance on Occupational Health Prevention (11), which are derived from the European Occupational Health and Safety Framework Directive 89/391/EEC (12). Generally speaking, OHP aims at improvements in the protection of the health of all employees by using findings regarding the causes of occupational diseases as a basis for improvements in working conditions. Regarding UVR, the focus is on advising workers on exposure and the resulting hazards to their skin and eyes. If physical or clinical examinations are not necessary or are refused by the employee, OHP is limited to a counseling interview.

With regard to exposure to solar UVR during outdoor activities, there is potential for improvement both in the context of prevention, for example in OHP, and with regard to the reporting and compensation of occupational diseases in many countries in Europe and the entire world. Germany has enacted legislation for both which may serve as proposals for the international community (13, 14). OHP must be offered to every employee in Germany whose activities meet certain criteria. Regarding UVR exposure, these include assessing the exposure period of the months from April to September, and the daily period from 11 a.m. to 4 p.m. (CEST). If an employee has worked outdoors for more than 1 h on more than 50 days during this period, he or she must be offered OHP. Thus, a distinct definition of outdoor workers at risk from solar UVR has been established by the German Federal Ministry of Labor and Social Affairs. To my knowledge, this is the first country in the world to do so. This legislation is backed up by extensive measurements of the actual UV exposure of workers in Germany (15).

NMSC as a recognized occupational disease is not widespread either in Europe or in the world (16). The epidemiology required often suffers from the fact that cancer registries do not report these types of cancer, or the data sets are qualitatively questionable or incomplete. Many cases are also not reported, resulting in a significant underestimation of incidence (17). In Europe, it has therefore been proposed that cancer registries in particular take up this special focus on cancers that have an identifiable, preventable risk factor as their cause, such as occupational UVR exposures (18).

This also proves the need for increased efforts in prevention with regard to the overall incidence and prevalence of these cancers. In Germany, the incidence of invasive SCC and BCC are in men 184.1 and 143.0 in women per 100,000 persons, respectively (19); *in situ* forms of cutaneous SCC, such as actinic keratoses or Bowen's disease are not included in these numbers. In Italy there is also the possibility of recognizing UVR induced skin cancer as an occupational disease. There, however, the reports are clearly below the rate that one would expect due to the geographical location. In the Trentino region, for example, an incidence of 61.5 was calculated for BCC and 16.3 for SCC, each per 100,000 citizens (20). But even there, the incidences are constantly rising, and it must also be considered that the prevalence of NMSC is higher in the south of the country than in the north (21, 22).

In the new and upcoming ICD-11, a distinction is made between the different entities of NMSC, so that a statistically reliable recording is possible through appropriate coding (23).

Experience with the occupational disease in Germany since its introduction in 2015 has proven the high incidence of these diseases. So far—cumulatively from 2015 to 2019—~44,000 occupational disease reports have been filed with ~60% being recognized. It is inconceivable that this number should be lower in other countries, especially in more southern countries, because of the higher radiation levels. Radiation levels directly depend on the solar inclination angle. Thus, the radiation is highest at the equator and lessens with increasing latitudie. For example, the erythemal active UVR level in Germany is only about 26% compared to the equator (24).

The aim of this work is to show which occupations and sub-occupations are affected according to scientifically based current legal criteria for OHP, as this is unknown so far. There is a direct applicability for other nations from the underlying data and findings and the fact that Germany in particular is a country located in higher latitudes. These data coupled with employment figures and economic directories allows the estimation of the number of people affected and the associated expenditure for the industry. According to known criteria, in this paper it is examined if the criteria are good for protecting against severe skin damage leading to an occupational disease.

## Methods

### Recording Radiation Exposure

**Figure 1:**
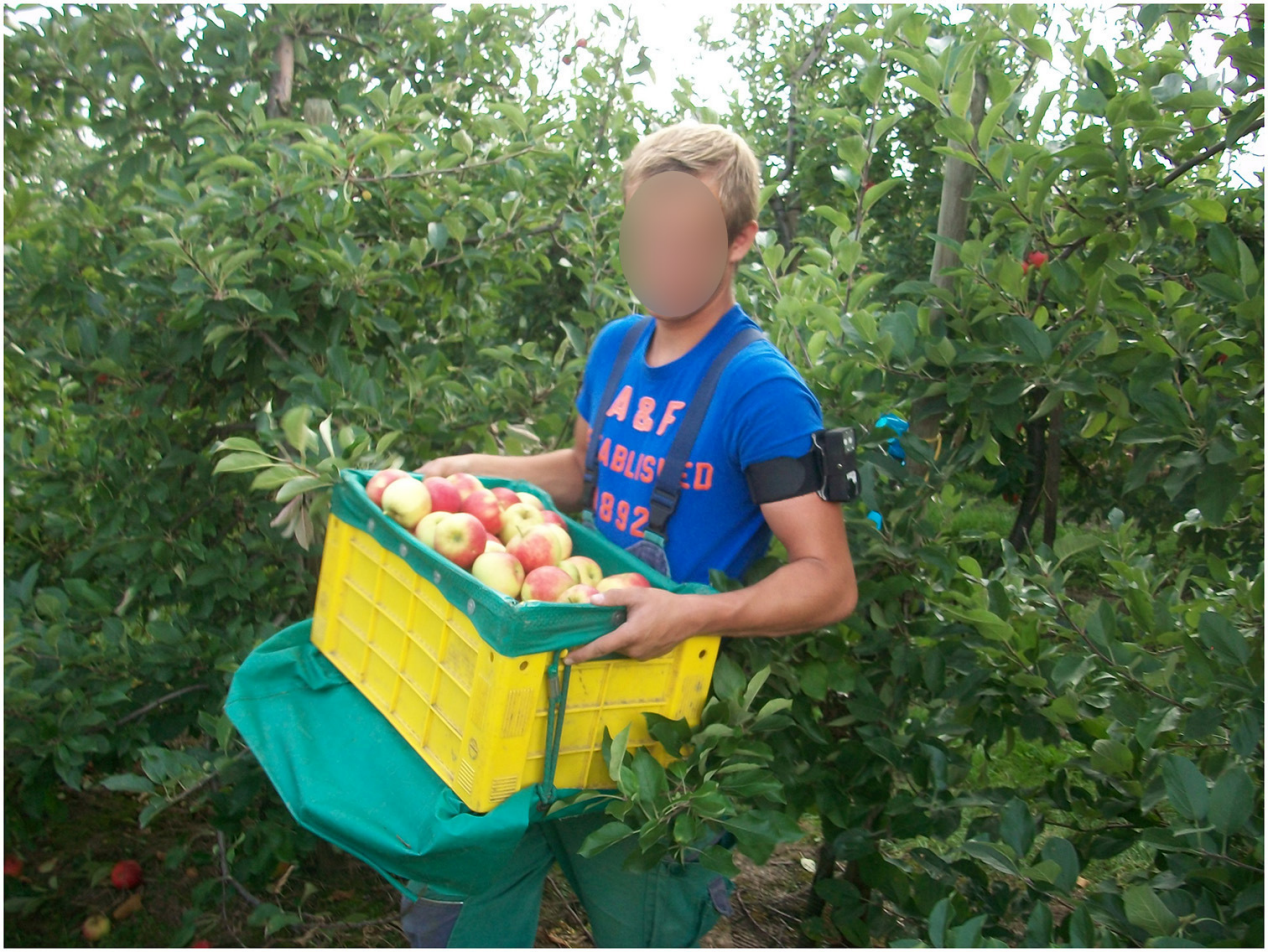
Photograph of a test person at work. The dosimeter was worn by the subjects on the left upper arm as standard (Image/IFA).

**Figure 2:**
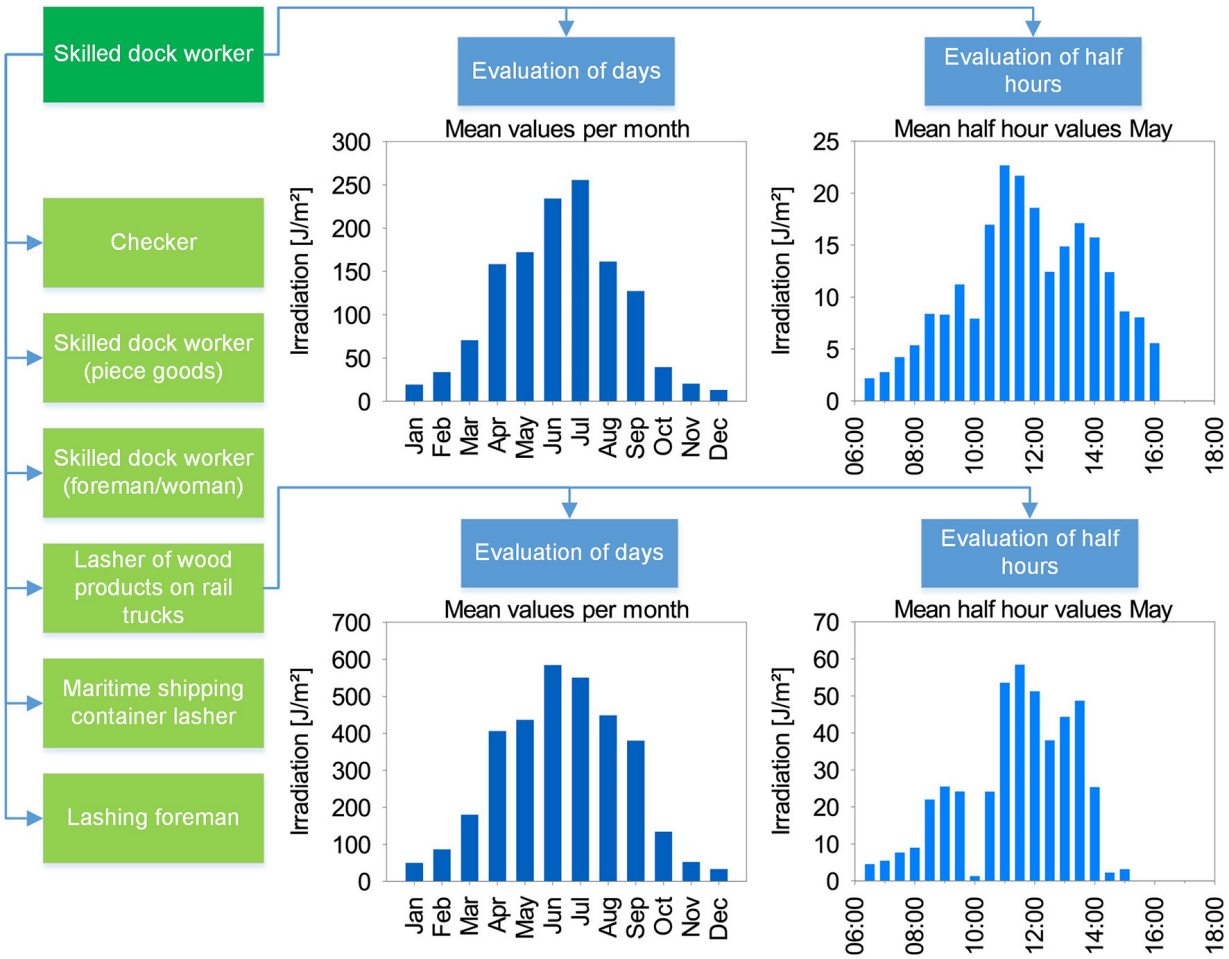
Data structure in GENESIS-UV using the example of skilled dock workers. Several sub-occupations (light green) can be assigned to one occupation (dark green). Detailed data is available in each case, giving a monthly daily average, as well as the daily distribution in half-hourly values, each month-related (here: example from month May).

With the GENESIS-UV (15) project (GENeration and Extraction System for Individual expoSure), personal dosimetric measurements of UVR during occupations since 2014 were performed. Each of the 1,000 test persons was equipped with a data logger dosimeter to conduct measurements every working day for seven months from April to October (see Figure 1). It was possible to collect information on more than 250 occupations and sub-occupations. Sub-occupations summarize concrete activities of employees that are too vaguely defined in the superordinate occupation [e.g., according to ISCO (25)]. For example, the occupation of gardeners subdivides into ornamental gardeners, cemetery gardeners, and several others. These data are currently being published. Activity profiles are assigned to each occupational context, which allow a precise identification of the individual activities. In addition to cumulative values and their statistical basis, individual daily doses of UV exposure can be presented for each occupation as well as for each test person. Furthermore, the measured values measured with GENESIS-UV every second can also be aggregated to half-hourly values. This plays a decisive role in the analysis of the criteria for OHP in this paper. An example of the structure of the available data is shown in Figure 2.

Since 2020, the exposure during leisure time activities is currently determined with GENESIS-UV. More than 500 test persons have been active over seven months so far. With the help of time use information for the population from the Federal Statistical Office, the average exposure of the population (or even individual groups) can be determined in detail and validated. Time use information describe the fraction of time which is spent for a distinct activity as a fraction of a 24-h-day. All activities for a group of people are included, e.g. such as sleeping, work, family time, sports, media use. This information is available down to a minute level. If such time use information is also known for other countries, then these results can be applied directly. At present, a surprisingly high average exposure of the population in Germany of 260 SED [SED, 1 SED = 100 J/m^2^ erythema-effective irradiation; corresponds to about one half sunburn dose for skin type I on the Fitzpatrick scale (3)] per year is already emerging. This provisional value is used for modeling in this paper.

We have developed a new overall metric based on personal dosimetric measurements, which is currently being published (26, 27). With this, a holistic overall view of all exposures in connection with solar UV radiation is possible.

### Patient and Public Involvement

No patients were involved. The test persons were acquired with the support of German social accident insurance institutions, which are in close contact to enterprises of their branch. First of all, occupations were selected that were associated with a supposedly high UV exposure. In this field, the potential test persons were then directly approached and recruited based on their willingness to participate. The measurements, which took place exclusively during daily working hours in the period from April to October, were compensated with an expense allowance.

### Criteria Analysis for Occupational Health Prevention

According to the legally anchored scientific opinion, OHP must be granted to every person who was active outdoors for more than 1 h between 11 a.m. and 4 p.m. (CEST) on more than 50 days in the period from April to September. If one calculates a quota from this, it is about 40% of the working days (20 working days in April, June, and September, or 21 working days in May, July, and August, respectively).

In Germany, exposure-risk relationships have been described for the risk-based concept in handling carcinogenic agents since 2005. Health-based occupational exposure limits often cannot be derived for carcinogenic agents because there is usually no exposure at which an adverse health effect on workers can be completely ruled out (28). The establishment of substance-specific exposure-risk relationships makes it possible to derive acceptance and tolerance concentrations associated with a defined, additional cancer risk. Thus, a risk is assigned to an exposure at the workplace (quantity/m3) based on an 8-h working day. According to the German law, a risk of 4/1,000 new cancers is tolerated and must not be exceeded. This concept can also be transferred to the risks associated with physical agents. The attempt to quantify the risk of skin cancer resulting from UVR exposure serves to compare work-related risks and is based on the data currently available. To simplify matters, this calculation is based on a linear dose-response relationship, although the relationship between UVR and SCC could be described with an exponential function.

The incidence rate of SCC in Germany is in the range of 100/100,000 (=1/1,000), as described in the introduction. In order to estimate the tolerance risk, the legally binding scientific justification for skin cancer as occupational disease is taken as a basis. Considering the annual and daily cycle of the UVR exposure, only the period from April to September (northern hemisphere) and the time interval 10:00–15:00 o'clock, respectively, are relevant. This time period covers 88% of the annual and 75% of the daily UVR (24).

Calculations lead to the point that an exposure of 1 h in the period mentioned above is below the tolerance risk and thus fulfills the exposure-risk relationship (29). Longer exposure leads to higher risk and will exceed the tolerable risk level. This finally is the rationale to chose 1 h per day as criterion for OHP that must not be exceeded.

Conversely, this expert opinion also states that 1 h of UV exposure per day is tolerable, regardless of the occupation. The occupation/sub-occupation that shows the highest exposure in 1 h thus defines the tolerable upper limit. This can be a different occupation/sub-occupation in each month, since the exposure strongly depends on the individual activities. From our database, for each month, the occupation/sub-occupation with the highest UV exposure in 1 h is searched and set as the tolerable reference limit (see Table 1A). In the next step, for every occupation/sub-occupation the number of days per month where daily irradiation is above this reference limit is counted (example see Table 1B). The sum of these days from April to September is divided by the total number of measurement days in the respective occupation/sub-occupation. The result is a ratio that indicates the proportion of employment days in the occupation/sub-occupation that are above the limit. If this exceeds 40%, OHP is required according to the criterion defined above.

**Table 1A:** Occupation and sub-occupation with the hightest exposure in 1 h per month.

**Month**	**Occupation**	**Sub-occupation**	**Timeslot [CEST]**	**Value [J/m^**2**^]**
April	Cable fitter or linesman	Electrical fitter (e.g., electronics technician for power plants)	13:00–13:30 14:00–14:30	88.78
May	Service fitter, wind farm technology	Rotor blade maintenance on wind turbines	12:30–13:00 13:30–14:00	98.21
June	Facade construction worker	Roof builder	11:30–12:00 13:30–14:00	109.31
July	Construction machine operator	Construction machine operator and canal/sewer/drain engineering worker	13:00–14:00	95.82
August	Overhead line worker/technician	Overhead line worker/technician	13:30–14:30	117.29
September	Elevation platform operator	Elevating platform operator	13:00–14:00	91.38

*All other occupations show lower hourly exposures within the respective month*.

**Table 1B:** Example for determination of quota for OHP.

	**Days above level**						**Totals**		
**Occ./Sub-Occ**.	**Apr**	**May**	**Jun**	**Jul**	**Aug**	**Sep**	**Sum**	**#MMD**	**Quota OHP [Ratio %]**
Bricklayer	83	110	112	130	74	100	609	782	78
Roofer	303	353	349	351	255	310	1,921	2,243	86
Kindergarten teacher	81	122	91	114	51	43	502	1,755	29

*The number of days above the tolerable reference limit is counted for every occupation and sub-occupation. Next, the sum of these numbers is set into relation to the total number of measurement days in that respective occupation/sub-occupation (#MMD). The result is the quota OHP in %*.

To carry out this analysis, a total of 45,000 measurement days were available across all occupations and sub-occupations.

**Figure 3:**
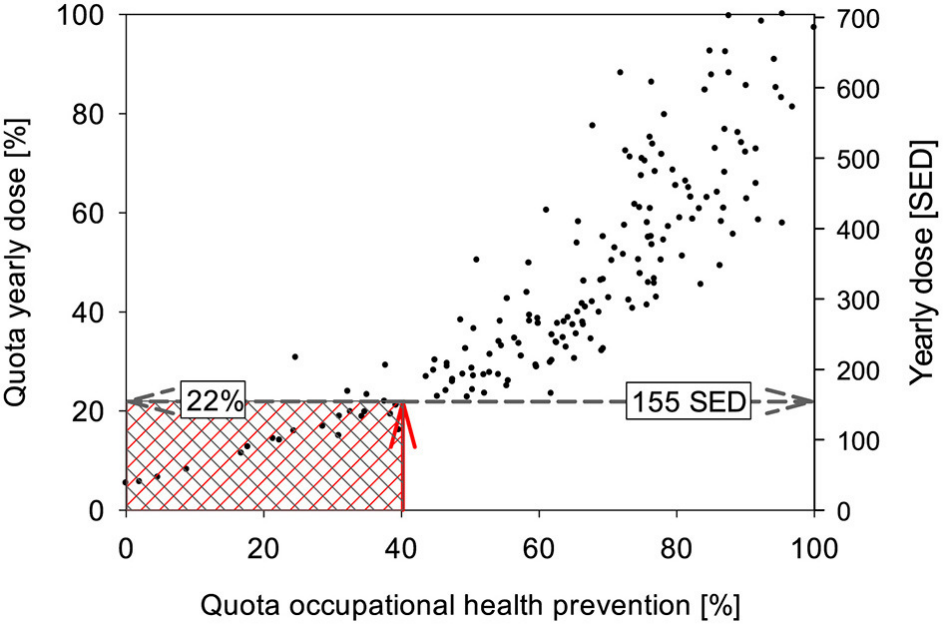
Relationship between the rate of days exceeding criteria (“OHP quota”) and the annual irradiation. The red arrow indicates the position of the criterion and its mapping to the distribution of the data, the gray arrow the mapping to the axes of the annual irradiation and its rate. Occupations/sub-occupations in the red shaded area do not meet the criteria for OHP, occupations/sub-occupations in the gray area are below the OD recognition criterion. The ranges are identical here, but may differ in case the criteria are defined differently.

The annual exposure values are available for all occupations and sub-occupations. If one defines that the occupation/sub-occupation with the highest annual exposure has been full-time exposed, then one can relate all other occupations to this and obtain information about the proportion of the occupation that takes place outdoors. In a diagram, this “quota” can be plotted against the “quota” from the proportion of days above the limit for OHP described above. A diagram is obtained which describes how the exceeding of the precautionary criterion is related to the annual irradiation. From the carry-over at the position of the 40% criterion from the OHP, the corresponding annual exposure can be derived (see Figure 3).

If one knows which occupations/sub-occupations are included, it is possible to estimate the number of people affected due to the variety of occupations investigated in the GENESIS-UV projects. For this purpose, the number of employees from the classification of occupations of the German Federal Employment Agency is accounted if this occupation is above the criterion of 40%. This results in the total number of people affected in Germany. This can also be directly transferred to the European Community via the NACE Rev.2 database (30). Due to the imprecise information on occupational fields, only a rough estimate based on ILO data can be made for the entire world (31).

### Reference to Occupational Disease Incidence

So far, there is no direct link between the prospective effect of OHP and the retrospective view of occupational diseases. Since January 2015, legal regulations have been in force in Germany that allow recognition and compensation for SCC and AK, under certain conditions (13, 32).

To date, no fixed irradiation dose could be found that can be used as a threshold for the development of SCC. Apparently, there is a relative measure which depends on the irradiation during the year. The more UVR irradiation occurs, the higher the number of cases of SCC and AK (33).

In German legislation on occupational diseases, it has become a good standard to assess diseases without a concrete trigger threshold via the epidemiologically derived doubling of the risk of disease. Although the number of studies on this was relatively small, it was possible to find a relation. An increase of 1% UVR leads to 2.5% more cases of SCC (34). It was concluded that 40% more UVR leads to a doubling of risk (100%).

This defines the demarcation criterion between an occupational disease and the widespread disease: a superadditive dose-response relationship, given 40% extra occupational exposure of the “normal” lifetime exposure, can give rise to an additional 100% risk due to occupation. If this legal and epidemiological framework is fulfilled, then the disease is considered to be occupationally caused. Although this criterion is legally specific only to Germany and Denmark, it forms a reliable and comprehensible basis for overall. Each person is assigned an average annual irradiation of 260 SED, which, after multiplication by age at initial diagnosis, yields the so-called “normal” lifetime irradiation (24).

In a hypothetical but typical case of a 65-year-old employee with 45 years of occupational outdoor work, the “normal” lifetime exposure calculates to be 16.900 SED, and thus the extra occupational dose is 6.760 SED to double the risk. Assuming longtime involvement with rather the same activities throughout the years, it can be calculated what average annual occupational exposure this person would have had to acquire in order to eventually meet the recognition requirement. This is 150 SED per year.

Ideally, OHP prevents an employed person from having to suffer an occupational disease or an occupational illness. By comparing to a hypothetical case of occupational disease as a basis, it can be estimated for the first time if the legal criterion chosen is suitable to do so. So far, there has not been a sufficiently large database of a physical or chemical agent to be able to make such a comparison.

Criteria of precaution must be suitable to prevent a later disease. Therefore, the two threshold values from the criterion for OHP and OD, respectively, must be comparable, ideally the value from OHP is much smaller. Then it could be concluded that a criterion has been found which has the goal of sparing the employee the fate of an occupational skin cancer disease. In addition, the affected occupations/sub-occupations would be identified and recorded.

## Results

The vast majority of the occupations and sub-occupations investigated so far exceed the threshold for the provision of OHP (Supplementary Table 1). Occupations from all sectors of the economy can be found above the relevant 40%. It is interesting to note that some employees who are known to work more than 1 h outdoors do not meet the criterion according to this analysis, for example educators or parts of forestry workers. Conversely, however, employees who were not previously in the focus, such as professional drivers in freight transport, surveyors or warehouse and transport workers, come into consideration. Viewed in a different way, the resolution of the data gives an indication that the breakdown of occupations into sub-occupations is of great importance in determining occupational safety and health measures, including OHP. In this way, sub-occupations can be identified for which the provision of OHP is not necessary (example: tower crane operators in the group of construction machinery operators), or is necessary in contrast to the occupations (example: workshop workers in the occupation of agricultural machinery mechanics). Of the 176 occupations and sub-occupations selected for this work, 153 (=87%) are so strongly associated with exposure that special OHP must be implemented. From the context in Figure 3 it follows that all occupations with an annual exposure of more than 155 SED require OHP.

Based on the federal German employment figures, the total number of affected persons can be extrapolated for Germany (see Table 2). According to this, about 7.2 million employees are eligible for OHP. If this is put in relation to the total number of employees of about 45 million, this makes up a share of about 16%. Up to now, a much smaller share had been assumed in Germany, namely about 5%. The transfer to the European level succeeds by assuming that employment in the economic sectors is on average similar to Germany. Therefore, for the 28 member states of the European Union (EU-28, 2019) with a total number of 225.7 million employed persons (quotation from Eurostat), one can estimate that about 36.1 million employees would be affected.

**Table 2:** Number of employees affected in Germany by exceeding OHP criteria based on official German Federal Statistical employment data of the different sectors.

**Industry sector in Germany**	**# Employees affected**
Agriculture	400,979
Animal husbandry	33,798
Occupations in the horse industry	13,104
Occupations in the horse industry-horse breeding	500
Supervision and management—horse industry	463
Animal care	30,574
Viticulture	3,939
Forestry, hunting, landscape management	48,119
Horticulture	381,094
Mining, open-cast mining, blasting	23,244
Natural stone and mineral processing occupations	13,498
Woodworking and wood processing occupations	82,107
Production of wood-based materials and components	10,185
Occupations in wood, furniture, interior construction	154,087
Metal construction occupations	284,751
Supervision—metal construction and welding	10,238
Occupations in renewable energy technology	7,206
Occupations line installation, maintenance	20,755
Structural and civil engineering occupations	745,438
Screed and terrazzo laying occupations	4,765
Painters, plasterers, building sealers, building protection	187,849
Dry construction, iso-room-glass roll construction	187,092
Supply and disposal	207,904
Warehousing, postal services, delivery, cargo handling	2,706,416
Vehicle guidance in road traffic	1,500,570
Construction and transport equipment management1	114,600
Sports instructors	45,992
Sum	7,219,267

A further step is extrapolation to the global level, but this can only be an estimate. Significantly different distribution of economic sectors in the specific countries, different behavior, also with regard to exposure, informal work make the extrapolation imprecise. Assuming 3 billion employees worldwide (global workforce) and transferring the quota of those affected from the EU, this results in a number of 480 million employees. However, as occupational health and safety standards are lower in many countries and the number of employees in the agricultural, construction and raw materials extraction sectors is higher, a significantly higher number of people affected can be expected. A more detailed information can be elucidated from the ILO Legal Database on Industrial Relations (IRLex) [ILO, Geneva (www.ilo.org/irlex)] by comparing countries on legislation and else and the labor force statistics of the Organization for Economic Co-Operation and Development (OECD) worldwide (https://stats.oecd.org).

This is to compare to the average annual irradiation a person would have to acquire in order to be exposed to twice the risk of disease compared to the average population, as explained in detail in the Methods section. At the age of 65, a person living in the middle latitudes would receive an average lifetime irradiation of 16,900 SED, to which 6,760 SED would have to be added in order to double the risk. Equally distributed over 45 years of working life, this results in an average irradiation of 150 SED, which would have to be acquired at least annually.

## Discussion

Occupational health prevention for exposures to natural UVR is an important component in the prevention of UVR related skin cancers. Ideally, OHP prevents an employed person from suffering an occupational illness or disease. Up to now, there has been a lack of metrological and scientific proof as to which occupations or sub-occupations are particularly highly exposed and what effects can be expected on the subsequent occurrence of illness. This work solves this problem and for the first time brings OHP and OD into a metric context.

No studies can be found in the international literature that are based on an equally large sample of subjects and data. Measurements of exposure have often been performed using a technique that does not allow for day- or even hour-resolved analyses (35–37); also, there is a lack of breadth in the choice of occupational activities (38–41). None of the studies have analyzed the measured values with regard to occupational health issues, but have aimed exclusively to determine irradiation (42–45). Grandahl et al. (46) recently also performed detailed time-resolved recordings of exposure.

With this new metric, it was possible for the first time to define criteria of OHP regarding UV exposure based on measured values. The list of occupations and sub-occupations can be used directly in practice worldwide. The usefulness of this list becomes particularly clear when analyzing the individual economic sectors. While it is clear, as expected, that the construction sector is heavily affected, other sectors (agriculture, services, etc.) also contribute to the total of at least half a billion people worldwide (EU: 36 million, Germany: 7.2 million) who must be provided with effective prevention. According to the concept presented in this paper, the definition of “outdoor worker” can also be adressed. An outdoor worker is anyone who spends more than 22% of their working time outdoors (cf. Figure 3). The term outdoor worker has already permeated legislation and other bodies, so a clean definition is of great importance.

This study has limitations. As the data set was recorded in Germany, the transferability to other countries in terms of latitude has to be considered. However, this again tightens the criteria significantly, as UV irradiation increases towards the equator. Especially occupations that are now at the limit of the criterion will tend to be above it at lower latitudes. Comparative measurements of this is planned in other studies. In addition, the counting of individual days above the criterion may be subject to statistical fluctuations. Therefore, only occupations that showed at least 50 valid measurement days were selected for this study. In principle, the results obtained are subject to the problems of personal dosimetry measurements, but this was counteracted with a large number of subjects and an extremely high number of data sets (3.8 billion) and validation methods.

In principle, previously unrecognized occupational profiles may still be missing, but this can be inferred by analogy and expertise from the occupations studied so far in most cases.

Although the criteria for OHP and OD are only legally valid in Germany so far, they are based on the international state of science and can therefore be adopted for all nations and used as a basis for a scientific analysis.

Crucial to the success of OHP or prevention in general is its acceptance by workers, but also the conviction of those who are responsible and have to bear any costs. Since photodamage cannot be reversed but requires constant, lifelong aftercare and therapy, consistent and preventive occupational health and safety is of great importance. A future reduction in the burden of disease is currently being researched in systematic reviews initiated by the WHO and ILO (47). The return on prevention is obvious when one considers that simple measures of OHP and technical occupational safety are already sufficient to prevent serious and permanent medical interventions. For example, installation of shading (also in urban planning), reduction of time spent directly in the sun or wearing of long-sleeved clothing are simple, but very effective measures to reduce exposure. Therefore, it is a clear cost-benefit calculation in favor of prevention for both society and employers who have to pay into social security systems or provide direct compensation.

A further classification of these study results can be made by comparison with the exposure limit value of 1 SED per day (2, 3) proposed by WHO and ICNIRP, taking into account the vulnerable skin type I (1). From the selected criterion for OHP, an acceptable irradiation of 0.65 SED per working day can be derived, if one assumes an equal distribution of exposure over 230 working days per year. If the irradiation from leisure activities is also taken into account, this total irradiation is within the range of the proposed exposure limit value.

This work puts OHP for UVR exposure in a more concrete light. The high urgency for an enormously large number of people affected could be shown and leads to the realization that efforts in prevention must be significantly intensified worldwide. For the first time, it was possible to show a direct proof and connection between the criteria of OHP and possible future diseases. On the basis of this work, risk groups can be clearly identified, and given specific preventive care.

Special attention should be paid to the fact that occupational physicians, for example must be involved at an early stage. Medical doctors are already held in high esteem by people by virtue of their training, so that the content to be conveyed may have a better effect.

The insights gained in this work can be taken up by national and international organizations, interest groups and also legislators, as they allow direct implementation in regulations. Training curricula for the instruction of employees can be developed or updated according to the findings, in order to implement the aspirations of the WHO, the ILO and the EU outlined in the introduction. Consistency with the other measures of technical and behavioral preventive occupational health and safety, also and especially taking into account private exposures, would be an ideal, equally holistic approach to the prevention of skin cancer.

## Data Availability Statement

The original contributions presented in the study are included in the article/supplementary material, further inquiries can be directed to the corresponding author.

## Ethics Statement

Ethical review and approval was not required for the study on human participants in accordance with the local legislation and institutional requirements. The participants provided their written informed consent to participate in this study.

## Author Contributions

MW acted as sole author and performed the scientific analysis of the data.

## Conflict of Interest

The author declares that the research was conducted in the absence of any commercial or financial relationships that could be construed as a potential conflict of interest. The reviewer KG declared a past collaboration with the author MW to the handling editor.

## Publisher's Note

All claims expressed in this article are solely those of the authors and do not necessarily represent those of their affiliated organizations, or those of the publisher, the editors and the reviewers. Any product that may be evaluated in this article, or claim that may be made by its manufacturer, is not guaranteed or endorsed by the publisher.
