# Supplementary material for: Criteria for Occupational Health Prevention for Solar UVR Exposed Outdoor Workers-Prevalence, Affected Parties, and Occupational Disease
Source: Front Public Health. 2022 Jan 26;9:772290. doi: 10.3389/fpubh.2021.772290 (PMC8826221; doi:10.3389/fpubh.2021.772290)
Supplement: Supplementary file 1 [file Table_1.docx]

Supplementary Table 1: List of occupations/sub-occupations, ordered by criteria quota (left) or alphabetically (right). Occupations for which several sub-occupations already have been examined are also indicated as a whole. There is no indication of a sub-occupation in these rows. YD (Yearly dose) indicates the annual irradiation in SED.

| **Occupation** | **Sub-occupation** | **Quota [%]** | **YD [SED]** | **Occupation** | **Sub-occupation** | **Quota [%]** | **YD [SED]** |
| --- | --- | --- | --- | --- | --- | --- | --- |
| Dock worker | Lasher of wood products on rail trucks | 100,0 | 685 | Agricultural engineer | Plant husbandry and plant cultivation | 35,0 | 163 |
| Drilling machine operator | Drilling machine operator | 96,9 | 572 | Agricultural machinery mechanic, general |  | 34,7 | 139 |
| Facade construction worker | Roof builder | 95,4 | 704 | Agricultural machinery mechanic, general | Agricultural machinery operator | 32,6 | 139 |
| Aircraft ground handler | Aircraft ground handler on the airport apron | 95,4 | 407 | Agricultural machinery mechanic, general | Workshop worker | 40,1 | 138 |
| Canal/sewer/drain engineering worker | Canal/sewer/drain engineering worker | 95,3 | 585 | Agricultural specialist |  | 66,5 | 325 |
| Warehouse/store or transport worker | Order picker in the outdoor storage area (assembly parts) | 94,5 | 599 | Agricultural specialist | Trial field worker | 66,5 | 325 |
| Warehouse/store or transport worker | Timber yard worker (outdoors) | 94,2 | 639 | Agricultural/farm technician |  | 72,5 | 404 |
| Quarry worker | Quarry worker | 92,4 | 694 | Agricultural/farm technician | Technical agricultural work | 83,6 | 320 |
| Warehouse/store or transport worker | Tester (scrap acceptance) and sampler | 91,9 | 411 | Agricultural/farm technician | Fertilization and plant protection | 55,6 | 183 |
| Loader | Excavator/digger operator, wood products | 91,6 | 512 | Agricultural/farm technician | Research technician (agriculture) | 75,0 | 499 |
| Woodworker | Woodworker | 91,6 | 463 | Agrobiologist | Biological laboratory technician | 67,9 | 545 |
| Woodworker |  | 90,2 | 441 | Aircraft ground handler |  | 77,1 | 302 |
| Road construction worker | Road construction worker | 90,1 | 602 | Aircraft ground handler | Aircraft ground handler on the airport apron | 95,4 | 407 |
| Gardener, general | Garden, landscape and sports field builder | 90,1 | 508 | Aircraft ground handler | Cargo handler on the airport apron | 61,9 | 212 |
| Concrete worker | Concrete worker | 89,5 | 521 | Airport loadmaster |  | 44,9 | 213 |
| Elevation platform operator | Elevating platform operator | 89,0 | 536 | Airport loadmaster | Acting loadmaster on the airport apron | 69,4 | 229 |
| Woodworker | Woodworking mechanic (specialism: construction elements) | 88,2 | 391 | Airport loadmaster | Responsible loadmaster on the airport apron | 37,7 | 205 |
| Construction machine operator | Construction machine operator and canal/sewer/drain engineering worker | 87,6 | 620 | Blaster (excluding shotfirers) | Charge firer in open-cast mining | 74,5 | 355 |
| Service fitter, wind farm technology | Rotor blade maintenance on wind turbines | 87,6 | 701 | Boat builder | Boat builder | 59,6 | 205 |
| Market gardener |  | 87,1 | 650 | Bricklayer/mason | Bricklayer/mason | 77,9 | 504 |
| Roofer | Roofer | 87,0 | 540 | Builder or assembler of prefabricated wooden elements | Prefabricated house assembler | 76,5 | 376 |
| Groom, jockey or allied profession | Groom | 87,0 | 479 | Building inspector |  | 61,8 | 165 |
| Facade construction worker | Roof and facade construction worker | 86,9 | 428 | Cable fitter | Glass fibre cable fitter | 54,1 | 192 |
| Motor vehicle driver | Delivering cars on the company premises | 86,5 | 409 | Cable fitter or linesman/lineworker |  | 70,6 | 354 |
| Warehouse/store or transport worker | Order picker and skilled storeman in the construction materials trade | 86,4 | 347 | Cable fitter or linesman/lineworker | Electrical fitter (e.g. electronics technician for power plants) | 74,7 | 429 |
| Facade construction worker |  | 85,9 | 451 | Cable fitter or linesman/lineworker | Cable laying (laying buried cables) Civil engineering worker | 66,7 | 288 |
| Roofer |  | 85,6 | 513 | Canal/sewer/drain engineering worker |  | 76,4 | 607 |
| Seasonal agricultural labourer or fruit picker | Seasonal agricultural labourer or fruit picker primarily working in the field | 85,1 | 617 | Canal/sewer/drain engineering worker | Canal/sewer/drain engineering worker | 95,3 | 585 |
| Metalworker/fitter/locksmith | Machine/plant fitter/mechanic (pipeline construction) | 84,9 | 651 | Canal/sewer/drain engineering worker | Canal/sewer/drain engineering worker and construction machine operator | 71,9 | 620 |
| Postman/woman | Delivery by bicycle | 84,4 | 443 | Carpenter |  | 79,9 | 460 |
| Quarry worker |  | 84,2 | 596 | Carpenter | Carpenter | 76,9 | 480 |
| Agricultural/farm technician | Technical agricultural work | 83,6 | 320 | Carpenter | Carpenter and Roofer | 82,1 | 444 |
| Structural steel fitter | Steel construction worker – shed construction (foreman/woman) | 83,3 | 427 | Concrete worker | Concrete worker | 89,5 | 521 |
| Greeenkeeper assistant | Care of lawns and landscape areas | 82,4 | 413 | Construction labourer, other | Construction and repair work at the company's premises | 58,7 | 268 |
| Roofer | Roofer and carpenter | 82,1 | 444 | Construction machine operator |  | 69,4 | 388 |
| Carpenter | Carpenter and Roofer | 82,1 | 444 | Construction machine operator | Construction machine operator and canal/sewer/drain engineering worker | 87,6 | 620 |
| Loader | Pipe loader | 81,8 | 457 | Construction machine operator | Construction machine operator and pipeline constructor | 77,8 | 355 |
| Road construction worker |  | 81,3 | 467 | Construction machine operator | Construction machine operator and road construction worker | 75,9 | 387 |
| Gardener, general | Forestry nursery gardener | 80,9 | 360 | Construction machine operator | Tower crane operator | 4,6 | 46 |
| Facade construction worker | Roof and facade construction worker (foreman) | 80,5 | 415 | Dock worker |  | 54,4 | 268 |
| Carpenter |  | 79,9 | 460 | Dock worker | Checker | 63,5 | 244 |
| Gardener, general | Nursery gardener | 79,5 | 482 | Dock worker | Skilled dock worker (piece goods) | 47,5 | 185 |
| Hydraulic engineering worker | Wasserbauer(Streckenunterhaltung) | 78,9 | 402 | Dock worker | Lasher of wood products on rail trucks | 100,0 | 685 |
| Overhead line worker/technician | Overhead line worker/technician | 78,3 | 561 | Drilling machine operator | Drilling machine operator | 96,9 | 572 |
| Warehouse/store or transport worker |  | 78,1 | 383 | Elevation platform operator | Elevating platform operator | 89,0 | 536 |
| Bricklayer/mason | Bricklayer/mason | 77,9 | 504 | Excavator/digger operator | Foreman/woman in water resources management | 32,3 | 168 |
| Construction machine operator | Construction machine operator and pipeline constructor | 77,8 | 355 | Facade construction worker |  | 85,9 | 451 |
| Aircraft ground handler |  | 77,1 | 302 | Facade construction worker | Roof and facade construction worker | 86,9 | 428 |
| Carpenter | Carpenter | 76,9 | 480 | Facade construction worker | Roof and facade construction worker (foreman) | 80,5 | 415 |
| Showman | Amusement park worker | 76,8 | 321 | Facade construction worker | Roof builder | 95,4 | 704 |
| Gardener, general | Cemetery gardener | 76,8 | 328 | Farmer |  | 56,5 | 244 |
| Structural steel fitter | Steel construction worker – shed construction (incl. roof and facade) | 76,6 | 519 | Farmer | Vegetable growers (area larger than 100 hectare) | 44,7 | 198 |
| Builder or assembler of prefabricated wooden elements | Prefabricated house assembler | 76,5 | 376 | Farmer | Vegetable growers (area larger than 100 hectare), with livestock | 64,0 | 231 |
| Canal/sewer/drain engineering worker |  | 76,4 | 607 | Farmer | Vegetable growers (area smaller than 100 hectare), with livestock | 57,1 | 236 |
| Scaffold erector/scaffolder | Scaffold erector/scaffolder | 76,3 | 388 | Farmer | Transhumance (business owner and family members working in the business) | 65,7 | 409 |
| Structural steel fitter |  | 76,2 | 428 | Farmer | Demeter (biodynamic agriculture) businesses | 74,7 | 335 |
| Metalworker/fitter/locksmith |  | 76,2 | 529 | Farmer | Hop farmer | 50,5 | 257 |
| Gardener, general | Hotel gardener (BGN) | 75,9 | 323 | Fisher/fisherman | Inland fisherman | 59,9 | 272 |
| Construction machine operator | Construction machine operator and road construction worker | 75,9 | 387 | Forester (skilled forest worker) |  | 50,3 | 201 |
| Road construction worker | Road construction worker and construction machine operator | 75,9 | 387 | Forester (skilled forest worker) | Worker in a municipal/private forest | 47,5 | 182 |
| Road maintenance worker | Road maintenance worker (motorway) | 75,8 | 407 | Forester (skilled forest worker) | Forester | 64,9 | 263 |
| Skilled recycling worker | Recyclable materials and harmful substances depot attendant | 75,7 | 291 | Forester (skilled forest worker) | Nursery forester | 62,5 | 238 |
| Quarry worker | Quarry foreman/woman | 75,4 | 495 | Forester (skilled forest worker) | Forester (general) | 24,7 | 216 |
| Agricultural/farm technician | Research technician (agriculture) | 75,0 | 499 | Gardener, general |  | 72,3 | 362 |
| Winemaker, general | Winemaker primarily performing outdoor work | 74,9 | 474 | Gardener, general | Municipal landscape maintenance worker | 46,5 | 169 |
| Farmer | Demeter (biodynamic agriculture) businesses | 74,7 | 335 | Gardener, general | Arborist/tree climber | 73,6 | 286 |
| Cable fitter or linesman/lineworker | Electrical fitter (e.g. electronics technician for power plants) | 74,7 | 429 | Gardener, general | Nursery gardener | 79,5 | 482 |
| Blaster (excluding shotfirers) | Charge firer in open-cast mining | 74,5 | 355 | Gardener, general | Forestry nursery gardener | 80,9 | 360 |
| Hydraulic engineering worker | Hydraulic engineer (water body maintenance) | 74,0 | 433 | Gardener, general | Cemetery gardener | 76,8 | 328 |
| Gardener, general | Arborist/tree climber | 73,6 | 286 | Gardener, general | Garden, landscape and sports field builder | 90,1 | 508 |
| Machine/plant fitter/mechanic or repair fitter/mechanic | Machine/plant fitter/mechanic for the extraction or processing of natural stone | 73,3 | 501 | Gardener, general | Groundsman on the airport apron | 46,7 | 208 |
| Postman/woman | Mail and package delivery | 73,1 | 298 | Gardener, general | Hotel gardener (BGN) | 75,9 | 323 |
| Service fitter, wind farm technology |  | 72,6 | 509 | Gardener, general | Salesperson in the garden department of home improvement stores | 39,7 | 113 |
| Agricultural/farm technician |  | 72,5 | 404 | Gardener, general | Ornamental gardener | 50,5 | 190 |
| Gardener, general |  | 72,3 | 362 | Greeenkeeper assistant | Care of lawns and landscape areas | 82,4 | 413 |
| Canal/sewer/drain engineering worker | Canal/sewer/drain engineering worker and construction machine operator | 71,9 | 620 | Groom, jockey or allied profession |  | 58,6 | 276 |
| Hydraulic engineering worker |  | 71,1 | 372 | Groom, jockey or allied profession | Groom | 87,0 | 479 |
| Cable fitter or linesman/lineworker |  | 70,6 | 354 | Groom, jockey or allied profession | Riding instructor/horse-breaker | 39,2 | 148 |
| Hydraulic engineering worker | Dam observer | 70,2 | 301 | Helicopter pilot | Helicopter pilot on work flight (flight for visual inspection of gas lines) | 16,8 | 80 |
| Airport loadmaster | Acting loadmaster on the airport apron | 69,4 | 229 | Hydraulic engineering worker |  | 71,1 | 372 |
| Construction machine operator |  | 69,4 | 388 | Hydraulic engineering worker | Dam observer | 70,2 | 301 |
| Hydraulic engineering worker | Hydraulic engineering worker (dock maintenance) | 69,4 | 327 | Hydraulic engineering worker | Hydraulic engineer (German federal waterways management authority) | 55,4 | 300 |
| Locomotive switch helper | Shunter | 69,2 | 226 | Hydraulic engineering worker | Hydraulic engineer (water body maintenance) | 74,0 | 433 |
| Skilled swimming-bath worker | Skilled swimming pool worker >8 hours | 69,0 | 326 | Hydraulic engineering worker | Wasserbauer(Streckenunterhaltung) | 78,9 | 402 |
| Landfill worker | Maintaining bins | 68,8 | 280 | Hydraulic engineering worker | Hydraulic engineering worker (dock maintenance) | 69,4 | 327 |
| Agrobiologist | Biological laboratory technician | 67,9 | 545 | Landfill worker | Maintaining bins | 68,8 | 280 |
| Skilled swimming-bath worker |  | 67,8 | 295 | Loader |  | 65,5 | 379 |
| Specialist - Wasterwater technology | Sewage plant worker (multiple functions) | 67,6 | 242 | Loader | Excavator/digger operator, wood products | 91,6 | 512 |
| Cable fitter or linesman/lineworker | Cable laying (laying buried cables) Civil engineering worker | 66,7 | 288 | Loader | Pipe loader | 81,8 | 457 |
| Agricultural specialist |  | 66,5 | 325 | Loader | Loader in cement works | 2,0 | 40 |
| Agricultural specialist | Trial field worker | 66,5 | 325 | Locomotive switch helper | Shunter | 69,2 | 226 |
| Skilled swimming-bath worker | Skilled swimming pool worker <8 hours | 66,4 | 263 | Machine/plant fitter/mechanic or repair fitter/mechanic |  | 61,1 | 425 |
| Pipeline constructor | Pipeline construction worker | 66,4 | 266 | Machine/plant fitter/mechanic or repair fitter/mechanic | Machine/plant fitter/mechanic for the extraction or processing of natural stone | 73,3 | 501 |
| Shepherd or sheep farmer/rearer/breeder | Shepherd or sheep farmer/rearer/breeder | 66,3 | 293 | Market gardener |  | 87,1 | 650 |
| Farmer | Transhumance (business owner and family members working in the business) | 65,7 | 409 | Metalworker/fitter/locksmith |  | 76,2 | 529 |
| Refuse collection worker | Skilled recycling and refuse worker | 65,6 | 281 | Metalworker/fitter/locksmith | Machine/plant fitter/mechanic (railways) | 52,0 | 192 |
| Loader |  | 65,5 | 379 | Metalworker/fitter/locksmith | Machine/plant fitter/mechanic (pipeline construction) | 84,9 | 651 |
| Surveying assistant, no precise details | Surveyor's assistant | 65,4 | 250 | Mobile telephone systems fitter | Mobile telephone systems fitter | 48,6 | 269 |
| Postman/woman | Delivery on foot | 65,2 | 215 | Motor vehicle driver | Delivering cars on the company premises | 86,5 | 409 |
| Forester (skilled forest worker) | Forester | 64,9 | 263 | Overhead line worker/technician | Overhead line worker/technician | 78,3 | 561 |
| Road maintenance worker |  | 64,2 | 273 | PE teacher |  | 37,6 | 154 |
| Farmer | Vegetable growers (area larger than 100 hectare), with livestock | 64,0 | 231 | PE teacher | PE teacher 100% | 43,6 | 189 |
| Plant operator, general | Landfill attendant | 63,7 | 267 | PE teacher | PE teacher 50% | 34,3 | 132 |
| Dock worker | Checker | 63,5 | 244 | Pipeline constructor |  | 59,9 | 265 |
| Refuse collection worker |  | 62,7 | 265 | Pipeline constructor | Pipeline construction worker | 66,4 | 266 |
| Warehouse/store or transport worker | Depot worker in a fuel depot | 62,6 | 237 | Pipeline constructor | Pipeline constructor performing servicing tasks | 52,9 | 221 |
| Forester (skilled forest worker) | Nursery forester | 62,5 | 238 | Pipeline constructor | Pipeline constructor and construction machine operator | 51,0 | 355 |
| Aircraft ground handler | Cargo handler on the airport apron | 61,9 | 212 | Plant operator, general |  | 49,3 | 229 |
| Road maintenance worker | Road maintenance worker (municipal) | 61,9 | 248 | Plant operator, general | Conveyor, processing, crushing plant operator | 21,4 | 101 |
| Building inspector |  | 61,8 | 165 | Plant operator, general | Landfill attendant | 63,7 | 267 |
| Technical plant manager | Technical plant manager in the scrap metal trade | 61,7 | 209 | Postman/woman |  | 58,3 | 309 |
| Machine/plant fitter/mechanic or repair fitter/mechanic |  | 61,1 | 425 | Postman/woman | Delivery – parcel | 55,3 | 176 |
| Pipeline constructor |  | 59,9 | 265 | Postman/woman | Mail and package delivery | 73,1 | 298 |
| Fisher/fisherman | Inland fisherman | 59,9 | 272 | Postman/woman | Delivery by bicycle | 84,4 | 443 |
| Surveyor | Surveyor | 59,6 | 203 | Postman/woman | Delivery on foot | 65,2 | 215 |
| Boat builder | Boat builder | 59,6 | 205 | Pre-school teacher |  | 22,3 | 99 |
| Construction labourer, other | Construction and repair work at the company's premises | 58,7 | 268 | Pre-school teacher | Pre-school teacher, kindergarten | 28,6 | 118 |
| Groom, jockey or allied profession |  | 58,6 | 276 | Pre-school teacher | Pre-school teacher, pre-kindergarten | 8,8 | 57 |
| Structural steel fitter | Steel construction worker – mast erection | 58,5 | 350 | Professional driver (goods transport) |  | 52,9 | 195 |
| Postman/woman |  | 58,3 | 309 | Professional driver (goods transport) | Delivery driver in the beverages trade | 45,3 | 161 |
| Professional driver (goods transport) | Delivery driver in the food trade | 57,4 | 218 | Professional driver (goods transport) | Delivery driver in the beverages trade, primarily small customers, without order picking | 50,3 | 170 |
| Farmer | Vegetable growers (area smaller than 100 hectare), with livestock | 57,1 | 236 | Professional driver (goods transport) | Delivery driver in the food trade | 57,4 | 218 |
| Farmer |  | 56,5 | 244 | Quarry worker |  | 84,2 | 596 |
| Agricultural/farm technician | Fertilization and plant protection | 55,6 | 183 | Quarry worker | Quarry worker | 92,4 | 694 |
| Hydraulic engineering worker | Hydraulic engineer (German federal waterways management authority) | 55,4 | 300 | Quarry worker | Quarry foreman/woman | 75,4 | 495 |
| Postman/woman | Delivery – parcel | 55,3 | 176 | Refuse collection worker |  | 62,7 | 265 |
| Wagenmeister (rail transport) | Wagenmeister | 54,5 | 233 | Refuse collection worker | Skilled recycling and refuse worker | 65,6 | 281 |
| Dock worker |  | 54,4 | 268 | Refuse collection worker | Driver/binman | 38,5 | 135 |
| Surveying technician | Surveying technician | 54,2 | 239 | Refuse collection worker | Manual cleaning worker | 49,6 | 160 |
| Cable fitter | Glass fibre cable fitter | 54,1 | 192 | Road construction worker |  | 81,3 | 467 |
| Pipeline constructor | Pipeline constructor performing servicing tasks | 52,9 | 221 | Road construction worker | Road construction worker | 90,1 | 602 |
| Professional driver (goods transport) |  | 52,9 | 195 | Road construction worker | Road construction worker and construction machine operator | 75,9 | 387 |
| Traffic monitor / auxiliary policeman | Traffic warden | 52,1 | 165 | Road maintenance worker |  | 64,2 | 273 |
| Metalworker/fitter/locksmith | Machine/plant fitter/mechanic (railways) | 52,0 | 192 | Road maintenance worker | Road maintenance worker (motorway) | 75,8 | 407 |
| Pipeline constructor | Pipeline constructor and construction machine operator | 51,0 | 355 | Road maintenance worker | Road maintenance worker (municipal) | 61,9 | 248 |
| Farmer | Hop farmer | 50,5 | 257 | Roofer |  | 85,6 | 513 |
| Gardener, general | Ornamental gardener | 50,5 | 190 | Roofer | Roofer | 87,0 | 540 |
| Professional driver (goods transport) | Delivery driver in the beverages trade, primarily small customers, without order picking | 50,3 | 170 | Roofer | Roofer and carpenter | 82,1 | 444 |
| Forester (skilled forest worker) |  | 50,3 | 201 | Sawmill worker |  | 24,4 | 112 |
| Refuse collection worker | Manual cleaning worker | 49,6 | 160 | Sawmill worker | Sawmill manager | 31,0 | 133 |
| Plant operator, general |  | 49,3 | 229 | Scaffold erector/scaffolder | Scaffold erector/scaffolder | 76,3 | 388 |
| Service fitter, wind farm technology | Fitter/technician/master craftsman for wind turbines (onshore) | 48,9 | 192 | Seasonal agricultural labourer or fruit picker | Seasonal agricultural labourer or fruit picker primarily working in the field | 85,1 | 617 |
| Mobile telephone systems fitter | Mobile telephone systems fitter | 48,6 | 269 | Service fitter, wind farm technology |  | 72,6 | 509 |
| Dock worker | Skilled dock worker (piece goods) | 47,5 | 185 | Service fitter, wind farm technology | Fitter/technician/master craftsman for wind turbines (onshore) | 48,9 | 192 |
| Forester (skilled forest worker) | Worker in a municipal/private forest | 47,5 | 182 | Service fitter, wind farm technology | Rotor blade maintenance on wind turbines | 87,6 | 701 |
| Gardener, general | Groundsman on the airport apron | 46,7 | 208 | Shepherd or sheep farmer/rearer/breeder | Shepherd or sheep farmer/rearer/breeder | 66,3 | 293 |
| Stucco worker, plasterer or renderer | Stucco worker | 46,7 | 204 | Showman | Amusement park worker | 76,8 | 321 |
| Gardener, general | Municipal landscape maintenance worker | 46,5 | 169 | Skilled recycling worker | Recyclable materials and harmful substances depot attendant | 75,7 | 291 |
| Professional driver (goods transport) | Delivery driver in the beverages trade | 45,3 | 161 | Skilled swimming-bath worker |  | 67,8 | 295 |
| Airport loadmaster |  | 44,9 | 213 | Skilled swimming-bath worker | Skilled swimming pool worker <8 hours | 66,4 | 263 |
| Farmer | Vegetable growers (area larger than 100 hectare) | 44,7 | 198 | Skilled swimming-bath worker | Skilled swimming pool worker >8 hours | 69,0 | 326 |
| PE teacher | PE teacher 100% | 43,6 | 189 | Specialist - Wasterwater technology | Sewage plant worker (multiple functions) | 67,6 | 242 |
| Agricultural machinery mechanic, general | Workshop worker | 40,1 | 138 | Specialist restaurant employee or waiter/barman/bartender | Service at the tables and at the counter | 0,0 | 38 |
| Gardener, general | Salesperson in the garden department of home improvement stores | 39,7 | 113 | Structural steel fitter |  | 76,2 | 428 |
| Groom, jockey or allied profession | Riding instructor/horse-breaker | 39,2 | 148 | Structural steel fitter | Steel construction worker – shed construction (incl. roof and facade) | 76,6 | 519 |
| Refuse collection worker | Driver/binman | 38,5 | 135 | Structural steel fitter | Steel construction worker – shed construction (foreman/woman) | 83,3 | 427 |
| Airport loadmaster | Responsible loadmaster on the airport apron | 37,7 | 205 | Structural steel fitter | Steel construction worker – mast erection | 58,5 | 350 |
| PE teacher |  | 37,6 | 154 | Stucco worker, plasterer or renderer | Stucco worker | 46,7 | 204 |
| Agricultural engineer | Plant husbandry and plant cultivation | 35,0 | 163 | Surveying assistant, no precise details | Surveyor's assistant | 65,4 | 250 |
| Agricultural machinery mechanic, general |  | 34,7 | 139 | Surveying technician | Surveying technician | 54,2 | 239 |
| PE teacher | PE teacher 50% | 34,3 | 132 | Surveyor | Surveyor | 59,6 | 203 |
| Agricultural machinery mechanic, general | Agricultural machinery operator | 32,6 | 139 | Technical plant manager | Technical plant manager in the scrap metal trade | 61,7 | 209 |
| Excavator/digger operator | Foreman/woman in water resources management | 32,3 | 168 | Traffic monitor / auxiliary policeman | Traffic warden | 52,1 | 165 |
| Sawmill worker | Sawmill manager | 31,0 | 133 | Verification engineer or technical supervisory officer | SVLFG labour inspector (agriculture) | 17,7 | 89 |
| Warehouse/store or transport worker | Transport worker | 30,9 | 105 | Wagenmeister (rail transport) | Wagenmeister | 54,5 | 233 |
| Pre-school teacher | Pre-school teacher, kindergarten | 28,6 | 118 | Warehouse/store or transport worker |  | 78,1 | 383 |
| Forester (skilled forest worker) | Forester (general) | 24,7 | 216 | Warehouse/store or transport worker | Timber yard worker (outdoors) | 94,2 | 639 |
| Sawmill worker |  | 24,4 | 112 | Warehouse/store or transport worker | Tester (scrap acceptance) and sampler | 91,9 | 411 |
| Pre-school teacher |  | 22,3 | 99 | Warehouse/store or transport worker | Order picker in the outdoor storage area (assembly parts) | 94,5 | 599 |
| Plant operator, general | Conveyor, processing, crushing plant operator | 21,4 | 101 | Warehouse/store or transport worker | Order picker and skilled storeman in the construction materials trade | 86,4 | 347 |
| Verification engineer or technical supervisory officer | SVLFG labour inspector (agriculture) | 17,7 | 89 | Warehouse/store or transport worker | Depot worker in a fuel depot | 62,6 | 237 |
| Helicopter pilot | Helicopter pilot on work flight (flight for visual inspection of gas lines) | 16,8 | 80 | Warehouse/store or transport worker | Transport worker | 30,9 | 105 |
| Pre-school teacher | Pre-school teacher, pre-kindergarten | 8,8 | 57 | Winemaker, general | Winemaker primarily performing outdoor work | 74,9 | 474 |
| Construction machine operator | Tower crane operator | 4,6 | 46 | Woodworker |  | 90,2 | 441 |
| Loader | Loader in cement works | 2,0 | 40 | Woodworker | Woodworking mechanic (specialism: construction elements) | 88,2 | 391 |
| Specialist restaurant employee or waiter/barman/bartender | Service at the tables and at the counter | 0,0 | 38 | Woodworker | Woodworker | 91,6 | 463 |
